# Efficacy and safety of acupuncture in the treatment of the sequela of olfactory disorders after infection with COVID-19: A protocol for systematic review and meta analysis

**DOI:** 10.1097/MD.0000000000030844

**Published:** 2022-09-30

**Authors:** Chao Tang, Xiaoqin He, Wenkang Fu, Yaxin Du, Yuxin Huang, Lu Liu, Wanning Lan, Runjun Luo, Yue Liu

**Affiliations:** a The fifth Clinical Medical College of Guangzhou University of traditional Chinese Medicine, Guangzhou, China; b Clinical Medical College of Acupuncture and Rehabilitation, Guangzhou University of Traditional Chinese Medicine, Guangzhou, China; c The first Clinical Medical College of Guangzhou University of traditional Chinese Medicine, Guangzhou, China; d Dongguan Hospital of Guangzhou University of Traditional Chinese Medicine, Dongguan, China; e Guangdong Second Traditional Chinese Medicine Hospital, Guangzhou, China.

**Keywords:** acupuncture, COVID-19, meta-analysis, olfactory disorders, protocol, sequela, systematic review

## Abstract

From the end of 2019 to now, coronavirus disease 2019 (COVID-19) has put enormous strain on the world’s health systems. As a characteristic sign of COVID-19 patient, olfactory dysfunction (OD) poses considerable problems for patients. In China, acupuncture has been widely used to treat OD caused by COVID-19, but there is still a lack of evidence-based medical evaluation. This study was designed to evaluate the effectiveness and safety of acupuncture for the treatment of COVID-19 OD.

**Methods:**

According to the retrieval strategies, randomized controlled trials on the acupuncture for COVID-19 OD were obtained from Cochrane Central Register of Controlled Trials, Embase, PubMed, Web of Science, the Chinese National Knowledge Infrastructure, the Chinese Biomedical Literature Database, the Chinese Scientific Journal Database and the Wanfang Database, regardless of publication date, or language. Studies were screened based on inclusion and exclusion criteria, and the Cochrane risk bias assessment tool was used to evaluate the quality of the studies. The meta-analysis was performed using Review Manager (RevMan 5.3) and STATA 14.2 software. Ultimately, the evidentiary grade for the results will be evaluated.

**Results:**

The results of this meta-analysis will be submitted to a peer-reviewed journal for publication.

**Conclusion:**

This study will provide up-to-date summary proof for evaluating the effectiveness and safety of acupuncture for COVID-19 OD.

## 1. Introduction

In December 2019, Coronavirus Disease 2019 (COVID-19) outbreak occurred in Wuhan, Hubei Province, China and spread rapidly throughout China, and then emerged around the world.^[1–3]^ Clinical manifestations of COVID-19 range from mild, cold-like symptoms typically associated with respiratory tract infections, such as cough and fever, to severe pneumonia with respiratory failure.^[4]^ Frequently, patients also experience smell and taste disorders.^[5–11]^ Olfactory dysfunction (OD), including anosmia and hyposmia, manifests itself particularly prominently among these symptoms in COVID-19 patients.^[12]^ The ongoing COVID-19 pandemic has drawn attention to postviral OD (PVOD), which affects >50% of patients with severe acute respiratory syndrome coronavirus 2 infection.^[13]^ OD significantly impacts quality of life and has been associated with depression and an increased risk of future mortality.^[14–16]^ As the impact of COVID-19 continues to rise, we anticipate exponential growth in the numbers of patients seeking care for PVOD.^[17]^ Unfortunately, the treatment of PVOD is challenging.^[18]^ At present, olfactory training is recommended as a first-line therapy for the treatment of PVOD, with topical corticosteroids, sodium citrate, oral vitamin A, and TCA considered as optional therapies for appropriately selected patients.^[19]^ However, these treatments are sometimes ineffective. No high-level evidence are available to date on the efficacy of these measures in PVOD and further study is needed.

As an important part of external treatment of Traditional Chinese Medicine, acupuncture is widely used in the prevention and treatment of a variety of diseases.^[20]^ A large number of studies have proved that acupuncture has unique advantages in the treatment of OD and has been widely used in the world.^[21–23]^ During the COVID-19 outbreak, acupuncture was used as an adjunctive therapy for COVID-19 in China, and its efficacy in treating COVID-19 was confirmed under traditional protocols.^[24]^ Some studies have shown that acupuncture is effective for patients with OD refractory to standardized corticosteroid and olfactory training therapy.^[25]^ To date, there is no high-quality evidence that acupuncture treats COVID-19 OD. Therefore, we designed this study to better understand the efficacy and safety of acupuncture in treating OD with COVID-19.

## 2. Methods and analysis

### 2.1. Objectives and registration

This systematic review will aim to evaluate the effect and safety of acupuncture in the treatment of the sequela of olfactory disorders after infection with COVID-19. This systematic review protocol has been registered in the PROSPERO (CRD42022346942). We will follow recommendations outlined in The Cochrane Handbook of Systematic Review of Interventions and the preferred reporting items for systematic reviews and meta-analysis protocol (PRISMA-P) statement guidelines. If amendments are needed, we will update our protocol to include any changes in the whole process of research.

### 2.2. Eligibility criteria

PICOS principles will be consulted to establish the inclusion and exclusion criteria of this systematic review.

#### 2.2.1. Types of participants.

Patients who have been infected with COVID-19 and have developed PVOD (PVOD persists beyond 2 weeks of upper respiratory tract infection resolution^[26]^). There are no restrictions on gender, race, and stage of disease. The olfactory disorders caused by trauma history, toxic substance exposure history, mental factors and other factors are excluded. The diagnosis of COVID-19 and OD includes Chinese or international diagnostic criteria.^[27–29]^

#### 2.2.2. Types of interventions and comparators.

In addition to the treatment of COVID-19, treatment group interventions comprised acupuncture, and comparator groups intervention: comfort therapy (placebo, pseudo-acupuncture, or blank control), other therapies (Western medicine, usual care or non-drug therapy, etc).

#### 2.2.3. Types of outcomes.

In this meta-analysis, the olfactory function will be evaluated by the Sniffin’Sticks Test^[30]^ (Burghart GmbH, Wedel, Germany) and the main outcome is olfactory function test score. The secondary outcomes will assess quality of life and the incidence of adverse events.

#### 2.2.4. Types of studies.

This study will only include RCTs of acupuncture alone or combined with other interventions in the treatment of the sequela of olfactory disorders after infection with COVID-19. There are no restrictions on the publication language. Non-randomized controlled trials, case reports, clinical experience, and animal trials will be excluded.

### 2.3. Data sources and retrieval strategy

Randomized controlled trials will be extracted from Cochrane Central Register of Controlled Trials, Embase, PubMed, Web of Science, Chinese National Knowledge Infrastructure, Chinese Biomedical Literature Database, the Chinese Scientific Journal Database and the Wanfang Database. The set period was from the December 2019 to August 2022. All RCTs examining the use of acupuncture in the treatment of OD with COVID-19 will be collected. The main search terms will be “COVID-19,” “olfactory dysfunction” and “acupuncture,” and the retrieval formula will be adjusted according to the characteristics of different databases. Taking PubMed as an example, the retrieval strategy is shown in Table 1.

**Table 1:** Search strategy for the PubMed database.

#1 (covid 19[Title/Abstract] OR 2019-nCoV[Title/Abstract] OR coronavirus disease 19[Title/Abstract] OR 2019 novel coronavirus[Title/Abstract] OR coronavirus disease 2019[Title/Abstract] OR disease 2019 coronavirus[Title/Abstract] OR sars coronavirus 2 infection[Title/Abstract] OR SARS-CoV2[Title/Abstract])<?Char=Text?>
#2 (acupuncture[Title/Abstract]OR electro acupuncture[Title/Abstract]OR ear acupuncture[Title/Abstract] OR auricular acupuncture[Title/Abstract]OR ear needling[Title/Abstract]OR scalp acupuncture[Title/Abstract]OR head needle[Title/Abstract]OR head acupuncture [Title/Abstract]OR pyonex[Title/Abstract] OR plum-blossom needle[Title/Abstract]OR percussopunctator[Title/Abstract]OR eye needle[Title/Abstract]OR eye acupuncture[Title/Abstract])
#3 (olfactory dysfunction[Title/Abstract] OR Olfaction Disorders[Title/Abstract] OR Smell Disorder[Title/Abstract] OR Phantosmia[Title/Abstract] OR Smell Dysfunction[Title/Abstract] OR Olfactory Impairment[Title/Abstract] OR Dysosmia[Title/Abstract] OR anosmia[Title/Abstract] OR hyposmia[Title/Abstract] OR Smell Loss[Title/Abstract])
#1 and #2 and #3

### 2.4. Data collection and analysis

#### 2.4.1. Selection of studies.

**Figure 1:**
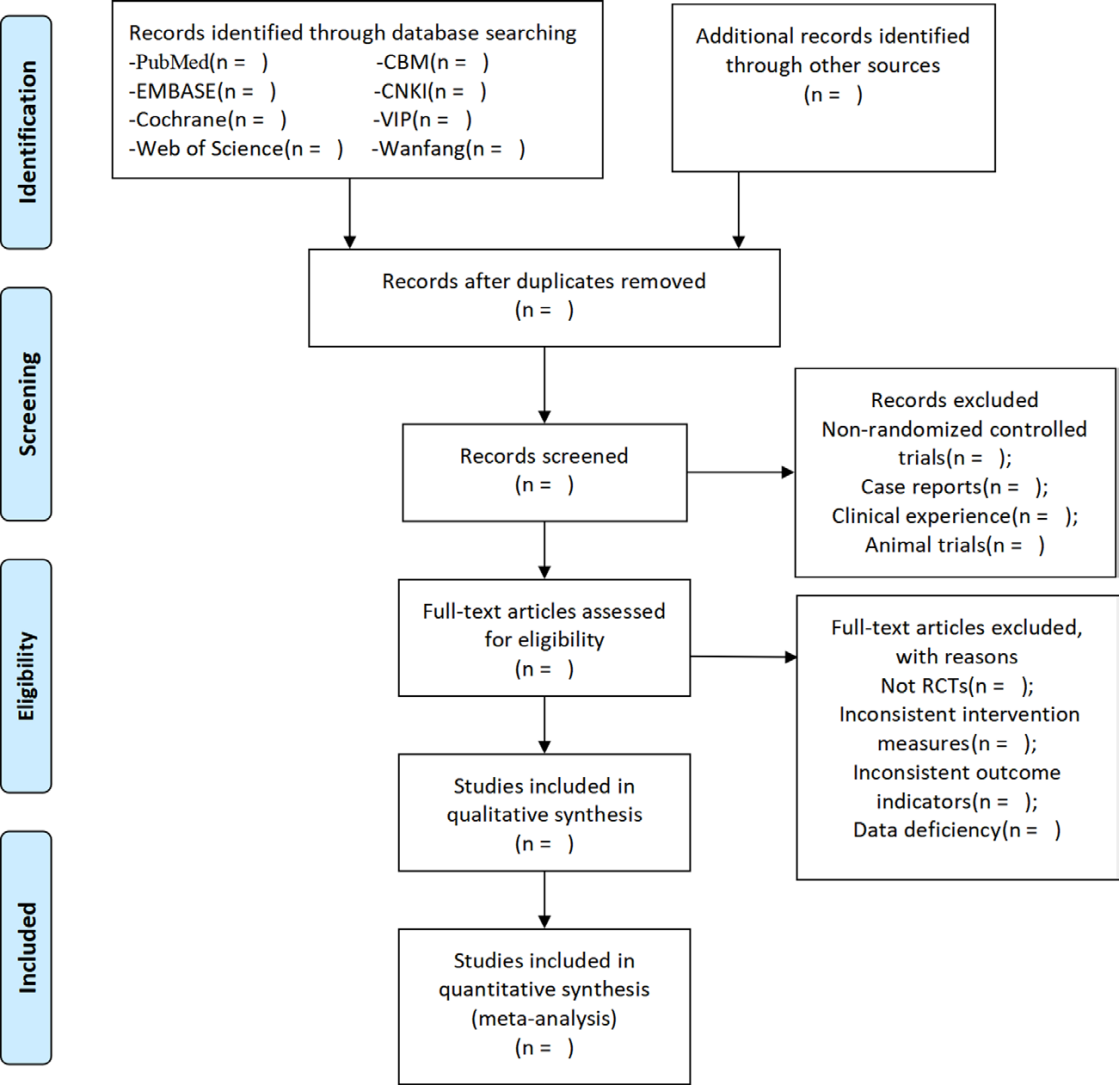
PRISMA flow chart of study selection process. PRISMA = preferred reporting items for systematic reviews and metaanalysis.

Two reviewers will search the study independently, and then they will screen the studies by reviewing titles and abstracts or the full text if necessary. Further, unresolved discrepancies will be managed by a third reviewer. The selection process was summarized using the PRISMA flow diagram. Details of the selection procedure for the studies are shown in the PRISMA flow chart (Fig. 1).

#### 2.4.2. Data extraction and management.

Two reviewers will be responsible for the extraction and management of data according to the retrieval strategy, including study title, journal, year of publication, name of first author, general information, study design, experimental intervention and timing of intervention, results, and adverse events. If there is a disagreement, the third researcher will be consulted.

#### 2.4.3. Dealing with missing data.

If complete literature or relevant data are not available, the corresponding author will be contacted. However, if missing data could not be obtained, the study was excluded from the analysis.

#### 2.4.4. Assessment of risk of bias.

Two researchers will use the risk of bias assessment tool recommended by Cochrane System Reviewer Manual 5.1.0 to evaluate the quality of the included RCTs. The following aspects will be considered: random sequence generation, allocation concealment, blinding of the participants and personnel, blinding of the outcome assessments, incomplete outcome data, selective reporting, and other sources of bias. These studies will be assigned as low risk, high risk, or unclear risk. Inconsistencies will be resolved by discussion with other reviewers.

#### 2.4.5. Measures of treatment effect.

Review Manager (RevMan 5.3, Cochrane Collaboration, Nordic Cochrane Center, Copenhagen, Denmark) software and Stata 14.2 (Stata Corp, College Station, Texas) will be used to conduct this metaanalysis. Dichotomous outcomes will be presented as risk ratios with 95% confidence intervals. When continuous outcomes exist, mean differences or standardized mean differences will be calculated.

#### 2.4.6. Assessment of heterogeneity.

Cochrane *X*^2^ and *I*^2^ tests will be used for the evaluation of heterogeneity. It is acknowledged that if *P* ≥ .05 and *I*^2^ ≤ 50%, the assessment of heterogeneity can be neglected; and there is great heterogeneity between included studies if *P* < .05 and *I*^2^ > 50%.

#### 2.4.7. Assessment of reporting bias.

If more than 10 articles are included in a certain outcome index, an inverted funnel chart will be used to analyze whether there is evidence of publication bias.

#### 2.4.8. Data synthesis.

We will take advantage of Review Manager (RevMan) software V.5.3 for data analysis and synthesis. Data will be processed with a fixed-effect model if no statistical heterogeneity was observed among the results (*P* ≥ .05 and *I*^2^ ≤ 50%). Meanwhile, the random-effect model will be put into use, if *P* < .05 and *I*^2^ > 50%.

#### 2.4.9. Subgroup analysis.

According to the results of the data synthesis, we will perform subgroup analyses or a metaregression to analyze the source of any heterogeneity.

#### 2.4.10. Sensitivity analysis.

Sensitivity analysis will be performed to examine the robustness of the study’s conclusions. Will include methodological quality, sample size, and the impact of missing data. Therefore, the impact of low-quality studies on overall results will be assessed.

#### 2.4.11. Quality of evidence evaluation.

The quality of evidence will be independently assessed by 2 reviewers and graded for recommendation evaluation, development and evaluation. Evidence quality will be rated as “high,” “medium,” “low,” or “very low” according to rating criteria based on 5 parameters (publication bias, inconsistencies, inaccuracies, and research limitations).

#### 2.4.12. Ethics and dissemination.

Since this study did not involve patient privacy, ethical approval was not required. Our research results will be published in peer-reviewed journals.

## 3. Discussion

COVID-19, caused by syndrome coronavirus 2, is a serious global public health threat that puts people around the world at risk.^[31]^ As a characteristic sign of COVID-19 patient, OD could influence severely the quality of life of affected subjects. However, the treatment of OD is challenging and the existing treatment with western medicine is limited. As an important part of external treatment of Traditional Chinese Medicine, acupuncture has unique advantages in the treatment of OD. Studies have reported that acupuncture may regulate the microcirculation under physiological or pathological conditions^[32]^ and significantly improve smell and taste functioning.^[33]^ Acupuncture may aid the treatment of PVOD patients who are refractory to drugs or other therapies^[25]^ and possibly offers a new therapeutic option for PVOD. Thus, this study will systematically evaluate the effectiveness and safety of acupuncture in the treatment of OD with COVID-19 and provide evidence-based medicine guidance for acupuncture in the treatment of OD with COVID-19.

## Author contributions

Runjun Luo and Yue Liu are the guarantor of the article and will be the arbitrator when meeting disagreements. All research members participated in developing the criteria and drafting the protocol for this systematic review. CT, XH and WF established the search strategy. XH,WF and YD will independently accomplish the study selection, data extration and assess the risk of bias. CT, LL and WL will perform the data syntheses. The subsequent and final versions of the protocol are critically reviewed, modified and authorized by all authors.

**Conceptualization:** Chao Tang, Xiaoqin He.

**Data curation:** Chao Tang, Xiaoqin He.

**Formal analysis:** Xiaoqin He, Wenkang Fu.

**Funding acquisition:** Wenkang Fu.

**Investigation:** Wenkang Fu.

**Methodology:** Wenkang Fu, Yaxin Du.

**Project administration:** Yaxin Du.

**Resources:** Yaxin Du, Yuxin Huang.

**Software:** Yuxin Huang, Lu Liu.

**Supervision:** Yuxin Huang, Lu Liu, Wanning Lan.

**Validation:** Lu Liu, Wanning Lan.

**Visualization:** Wanning Lan.

**Writing – original draft:** Chao Tang.

**Writing – review & editing:** Runjun Luo, Yue Liu.
